# Intrinsic Image Decomposition via Structure-Preserving Image Smoothing and Material Recognition

**DOI:** 10.1371/journal.pone.0166772

**Published:** 2016-12-16

**Authors:** Ali Nadian-Ghomsheh, Yassin Hassanian, Keyvan Navi

**Affiliations:** 1 Cyber Space Research Institute, Shahid Beheshti University, Tehran, Iran; 2 Computer Science and Engineering Department, Shahid Beheshti University, Tehran, Iran; City University London, UNITED KINGDOM

## Abstract

Decoupling shading and reflectance from complex scene-images is a long-standing problem in computer vision. We introduce a framework for decomposing an image into the product of an illumination component and a reflectance component. Due to the ill-posed nature of the problem, prior information on shading and reflectance is mandatory. The proposed method adopts the premise that pixels in a region with similar chromaticity values should have the same reflectance. This assumption was used to minimize the *l*_*2*_ norm of the local per-pixel reflectance gradients to extract the shading and reflectance components. To obtain smooth chromatic regions, texture was treated in a new style. Texture was removed in the first step of the algorithm and the smooth image was processed for intrinsic decomposition. In the final step, texture details were added to the intrinsic components based on the material of each pixel. In addition, user-assistance was used to further refine the results. The qualitative and quantitative evaluation on the MIT intrinsic dataset indicated that the quality of intrinsic image decomposition was improved in comparison with previous methods.

## Introduction

Intrinsic image components can be regarded as a set of images describing an image in terms of scene illumination, shape and reflectance of surfaces in the scene. Decomposing an image into its intrinsic components has a wide range of applications in industry. Eliminating the shading component provides illumination-free models that could be used for relighting [1], retexturing [2, 3], gray scale colorization [4], and reflectance editing [5].

The process of recovering shading and reflectance can be accomplished by two approaches: using a single image or multiple images. Employing depth information and image sequences for deriving intrinsic components have also been considered in many studies. It is also convenient to aid intrinsic image decomposition through user-assistance. The user specifies pixels that have constant illumination or reflectance in order to disambiguate illumination and reflectance [6, 7]. Additional information contributes to the improvement of intrinsic image decomposition. However, the required multiple images and depth information limit the general application of these methods.

Acquiring intrinsic components from a single image is a non-trivial task due to its ill-posed nature, and solving this problem remains an open challenge. To solve the intrinsic decomposition problem, it is necessary to obtain prior knowledge on reflectance and shading. Some approaches consider the global sparsity assumption by stating that each image is composed of a small set of chrominance values, and smooth variation of intensity is due to luminance changes which should be assigned to the shading component. These methods usually apply clustering algorithms to find regions with similar chromaticity values and utilize the information within each cluster to calculate the reflectance value.

This paper proposes a new framework for intrinsic image decomposition. Our approach applies several steps in order to obtain high quality shading and reflectance components from a single image. The approach is based on the assumption that regions with smooth intensity variations share the same material properties and have the same reflectance. Thus, the reflectance of a pixel can be obtained as a weighted function of a connected set of pixels (Ω) with similar intensity values. To find Ω for an input pixel, region growing was applied to ensure that Ω is connected and any change of intensity is smooth. To avoid ambiguity caused by texture, we treat texture differently from the preceding methods. Texture details were removed from the image and the smooth image was processed for intrinsic decomposition. Texture details were added to the reflectance or shading components based on the material of each pixel in the final stage. To evaluate the performance of our method qualitatively, the algorithm was tested on several natural-scene images to demonstrate the advantages of the proposed method. For quantitative evaluation, the MIT intrinsic dataset was considered and the results were compared with results of methods tested on this dataset.

The contributions of the paper towards solving the intrinsic image decomposition problem can be stated as below:

- Applying material recognition for handling fine texture.
- Calculating the reflectance of the input pixel based on its neighbor pixels obtained via region growing which results in reflectance values that are globally more preserved.
- Performing structural-preserving image smoothing for handling fine texture details and enhancing the performance of region growing.

The rest of the paper is organized as follows: Section 2 describes pervious work related to intrinsic image decomposition. In Section 3, the method for intrinsic image decomposition is explained and the advantages of the method is discussed. Section 4 shows the experimental results and Section 5 concludes the paper.

## Related Work

Intrinsic image decomposition methods which compute shading and reflectance components are briefly reviewed in this section. We divide the methods into three categories, Retinex-based methods, global sparsity assumption methods, and methods that use additional information other than the 2D image.

### Retinex-Based methods

In 1971, Land and McCann [8] proposed the Retinex algorithm. They assumed that Mondrian-like images have regions of constant reflectance where large illumination gradient indicates reflectance changes, and small gradients are caused by shading. Intrinsic images were then decomposed by integrating their respective derivatives across the input image. For methods such as simple Retinex which operate on grayscale images, the output reflectance image has the same chromaticity as the input image. Therefore, this approach struggles if the assumptions of white light and Lambertian surfaces are violated. However, using RGB information, makes the method more resilient against the violation of these assumptions [9].

Inspired by the work of McCann [8], Fun et al. [10] extended the Retinex algorithm for color images by assuming that shading variations does not alter chromaticity, and associated reflectance derivatives to significant chromaticity changes. Garces et al. [11] leveraged the correlation between reflectance and chrominance reported in [10] by detecting regions of similar chromaticity in the input image to approximate regions of similar reflectance. Other algorithms have been built upon the original Retinex algorithm [12–14]. However, the original algorithm outperformed all the other algorithms prior to 2009 when tested on the MIT intrinsic image dataset [15]. Tappen et al. [16] presented a system that used multiple cues for recovering shading and reflectance components. They trained a classifier based on color information to distinguish illumination changes caused by shading and reflectance. They applied Markov Random Fields to propagate the classification of areas in order to disambiguate regions where the local analysis delivered unsatisfactory results. In Tappen’s method, training a comprehensive classifier suitable for all range of shading and reflectance is exhaustive, and classifying pixels into shading and reflectance based on local evidence is not always easy. Methods based on the Retinex algorithm are intuitively simple and efficient but the mentioned pre-assumption on real scenes does not always hold.

### Global sparsity prior

Some recent methods assume the global sparsity prior on reflectance which suggests natural images are subjugated by a relatively small set of material colors [17]. Bell et al. [18] used K-means clustering to estimate distinct regions and employed Conditional Random Fields (CRF) for pixel labeling. Applying clustering algorithms to find regions with distinct reflectance in the image has some disadvantages. Firstly, there is no guarantee that pixels in each cluster form connected regions. This could violate the assumption made by Fun et al. [10]. Secondly, in the case of *c*^*0*^ and *c*^*1*^ discontinuity, shading smoothness assumption breaks and leads to undesired clustering of the input image. Bi et al. [19] proposed an *L*^*1*^ image transform for image flattening and used the flattened image to develop a pipeline for intrinsic image decomposition relying on probabilistic boosting trees for reflectance labelling. Utilizing the smooth image for clustering is more desirable compared to clustering the raw input image because of higher probability of associating regions of similar chrominance to reflectance. Rother et al. [14] introduced prior on reflectance values as being drawn from a sparse set of basis colors resulting in a random field model with global, latent variables and pixel-level output reflectance values. Solving the intrinsic decomposition by energy minimization problems has been frequently proposed. Shen et al. [7] suggested neighboring pixels have similar intensity values and therefore have similar reflectance. Their decomposition was formulated by minimizing an energy function with the addition of a weighting constraint to the local image properties. Barron et al. [20] presented a unified method to shape, shading, and reflectance estimation from a single image. Nevertheless, for scene-level images, their assumption about depth continuity does not hold, and unsatisfactory results are produced for such images. Finlayson et al. [21] proposed entropy minimization for calculating illuminant-free images. The invariant image was derived from the physics behind color formation in the presence of a Planckian light source, Lambertian surfaces, and narrowband imaging sensors. Shen et al. [22] suggested neighboring pixels with similar chromaticity share the same reflectance. Also they adopted the premise that natural images are dominated by a small set of material colors. They used multi-resolution analysis to enforce the local reflectance sparseness constraint at a global level. They further applied a total-variations-like cost term to take into account the global sparsity assumption.

### Multiple Images and Image sequences

Some techniques use a sequence of images or video streams for intrinsic image decomposition. While some of these methods use multiple images to find the intrinsic component for a single image, other methods try to find the intrinsic components throughout the entire input video stream.

Bonnel et al. [23] used a hybrid *l*_*2*_*-l*_*p*_ formulation that separates image gradients into smooth illumination and sparse reflectance gradients using look-up tables. They used a multi-scale parallelized solver to reconstruct the reflectance and illumination from these gradients while enforcing spatial and temporal reflectance constraints. They also used user-assistance for refining the results of the initial decomposition. More recently, Meka et al. [9] proposed a variational approach by solving an *l*_*2*_*-l*_*p*_ optimization problem to find the intrinsic image components. Their optimization problem includes local sparsity prior on reflectance, spatio-temporal reflectance consistency prior, reflectance clustering prior, and a data fitting term.

Laffont et al. [24, 25] uses a collection of images taken from a scene for intrinsic image decomposition. Assuming Lambertian surfaces, they use Multiview-stereo to produce an oriented 3D cloud point of the scene, from which, they derive relationships between reflectance values at different locations, across multiple views. They then identify reflectance ratios between pairs of points and infer constraints to optimize a coherent solution across multiple views and illuminations. Lee et al. [26] used both image sequences and depth information to extract intrinsic components from video. Their shading constraints enforce relationships among the shading components of different surface points according to the similarity of the surface orientation. Further, temporal constraints applied to video data allowed for handling view-dependent non-Lambertian reflections. Incorporating video sequences makes it possible to use spatio-temporal features to increase the accuracy of intrinsic image decomposition and further provides information to handle intense lighting conditions. However, requirement of additional image frames and depth cues limits the application of these method when only a single image is available for decomposition.

The main focus of this study is to obtain intrinsic image components from a single image. In summary, our method applies the following steps to extract the shading and reflectance components: To preserve the piecewise constancy of chrominance values, we separate the texture information and process the smooth image for intrinsic decomposition. In the next step, assuming Mondrian-like images, we achieve a sparse solution by minimizing an *l*_*2*_ norm of the local per-pixel reflectance gradients. In contrast to conventional methods that use fixed size patches, we apply region growing for choosing the local pixels used in the minimization process. In addition, we consider material recognition in our frame work. Through material recognition, texture that was removed in the initial step, will be added to either reflectance or shading component based on the material of each pixel. Finally we consider user brushes for assisting the minimization process. The addition of user brushes allows to disambiguate shading and reflectance at a global level by defining regions of the image that share either the same reflectance or shading information. This step will help to find regions with similar reflectance and shading information at a global level defined by the user.

## Our Method

Intrinsic image decomposition is an ill-posed problem and cannot be solved without prior information on reflectance and illumination. The proposed approach in this paper is built based on well-established assumptions on reflectance and illumination, suggested by Funt et al. [10] and the Retinex algorithm. The first assumption suggests that changes in reflectance are associated with changes in chromaticity, and the Retinex algorithm suggests that shading is smooth. Based on these assumptions, we employed the prior on shading and reflectance as an image region with smooth intensity variations indicates constant reflectance. Thus, the reflectance of pixel *p* can be represented by the weighted summation of pixels in a set Ω that contains *p*, whose members have similar intensity compared to *p*.

**Fig 1:**
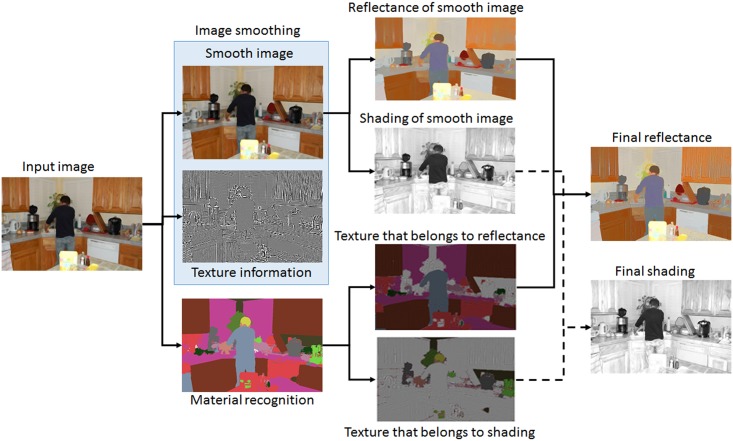
The proposed intrinsic image decomposition pipeline: The input image is processed for material recognition and image smoothing. The initial reflectance and shading components are extracted from the smooth image. The residue texture of the original image is assigned to either the shading or reflectance component based on the material of each pixel.

Implementation steps of our approach are depicted in Fig 1. First, a smooth version of the input image was obtained via structure preserving image smoothing. Then, the smooth version of the image was used for intrinsic decomposition, and the shading and reflectance components were extracted. In the final stage, the texture information was added to either the shading or the reflectance component based on the material of each pixel.

### Structure-preserving image smoothing

The proposed assumption on reflectance and shading suggests that pixels with similar chromaticity are parts of the same material and hence share the same reflectance values. Retinex algorithm [8] confirms the proposed assumption by suggesting that any change of intensity in an image is caused by either reflectance or shading, and generally, large gradients correspond to reflectance changes while shading is smoother. In some cases, the Retinex pre-assumption may not hold. For instance, the wrinkles on a piece of fabric produce large gradient changes that belong to the shading component. Therefore, removing texture details and processing the smooth image increases the chance of finding regions with similar reflectance values. In general, the image smoothing step has two objectives:

1. Handle fine synthetic texture: Many natural scene images contain fabric, tiles and similar regions with fine and smooth texture details that belong to the reflectance component. However, in many cases, variations of illumination may be assigned to the shading component. By image smoothing, it is possible to separate texture details which can be later reassigned to the shading or reflectance components based on the output of the material recognition algorithm.
2. Improving the calculated reflectance value: In order to estimate the reflectance of a pixel, we used the color values of its neighbor pixels which are found through region growing. Existence of small edges in the image reduces the performance of the region growing algorithm. In the smooth version of the image, larger regions can be found for calculating the reflectance value of the input pixel.

In order to find image regions with similar reflectance, it is necessary to remove as much shading information as possible without changing the image structure, i.e. obtaining the structure-preserved smooth version of the image. To acquire the smooth image, the method presented by Karacan et al. [27] was adopted. The desired goal is to decompose a given image *I* into its structural (*J*) and textural (*T*) parts as follows:

I=J+T (1)

The structural component of an image pixel is defined as:

$$J(p)=\frac{1}{\sum_{q}w_{pq}}\sum_{q\in N(p,r)}w_{pq}I(q)$$ (2)

where *N(p*,*r)* shows a square neighborhood centered at *p* with *(2r+1)*^*2*^ pixels. *w*_*pq*_ measures the similarity between *k*×*k* patches centered on pixels *p* and *q*. The similarity between two regions can be calculated via the region covariance descriptor proposed by [28], where an image region *R* is described with a *d*×*d* covariance matrix:

$$C_{R}=\frac{1}{n-1}\sum_{i=1}^{n}(z_{i}-\mu ){\left(z_{i}-\mu \right)}^{T}$$ (3)

**Fig 2:**
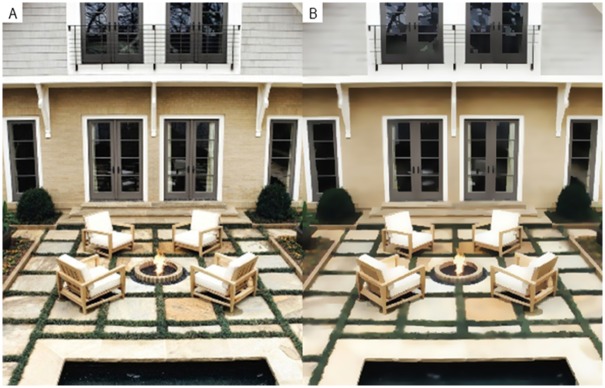
Structure-preserving image smoothing. (A) Original image, (B) smooth image (image selected from the MINC database [18] (S2 File)).

*z*_*i*_ = *1*,*…*,*n* denotes a d-dimensional feature vector inside *R* and *μ* is the mean of these feature vectors. The features are intensity values, first and second order intensity change in horizontal and vertical directions of the image, and pixel locations. Karacan et al. [27] proposed two functions for calculating the similarity between pixels. Based on our observations, the *w*_*pq*_ that better suits our approach is defined as:

$$w_{pq}=\text{exp}\left(\frac{-{\left(\sqrt{(\mu_{p}-\mu_{q})C^{-1}{\left(\mu_{p}-\mu_{q}\right)}^{T}}\right)}^{2}}{2\sigma^{2}}\right)$$ (4)

where *C = C*_*p*_ *+ C*_*q*_ and *μ* are region covariance and mean values extracted for image patches centered at *p* and *q*, respectively. Fig 2 illustrates the original image and the output smooth image. The edges of the image caused by shading have disappeared and smooth regions (Ω) can be easily detected from *J*.

### Intrinsic decomposition

In the second stage of our algorithm, the smooth image *J* is processed for intrinsic decomposition. The interaction between light and objects can be described using RGB color channels. Assuming Lambertian surfaces, the observed color at pixel of the input image can be denoted as:

J = S.×R (5)

*R* and *S* are the reflectance and shading components respectively, and × denotes per-channel multiplication. The objective is to retrieve S and R components for every pixel *i ϵ J*_,_ but Eq (5) is an ill-posed problem with two unknowns and one known which results in an infinite set of answers without any prior information on shading or reflectance. Lambert surface is the general assumption for solving many image processing problems. In the case of intrinsic image decomposition using a single image, since there is no information about lighting conditions and scene geometry, it is not possible to use more complex models and the Lambert assumption has been used frequently in previous methods [2, 6, 19].

In order to solve Eq (5), a new decomposition approach is developed based on the assumption that pixels of a connected region Ω with similar pixel intensity values have the same reflectance. Thus, *R* for a pixel *i ϵ Ω* is obtained as the weighted sum of reflectance values of pixels *p*_*j*_ *ϵ Ω*, denoted as:

$$R_{i}=\sum_{j\in \Omega }w_{ij}R_{j}$$ (6)

where *w*_*ij*_ defines the similarity between *p*_*i*_ and *p*_*j*_. In (6), we have followed the notation of Shen et al. [7], however, our approach for choosing Ω is different as will be explained in the next section. To measure similarity between pixels, many affinity functions have been proposed in the literature of image segmentation [24, 27, 29, 30] and colorization [31]. We considered two terms for measuring affinity between *p*_*i*_ and *p*_*j*_: illumination similarity and color angle difference. Illumination difference between *p*_*i*_ and *p*_*j*_ as a conventional affinity function is defined as $w_{ij}^{I}$ and formulated as:

$$w_{ij}^{I}=\text{exp}\left(\frac{-{\left\|Y_{i}-Y_{j}\right\|}_{2}^{2}}{2\sigma_{y}^{2}}\right)$$ (7)

where Y is the luminance component of a pixel in YUV color space and *σ*_*y*_ represents the variance between the luminance of pixel *i* with its neighbor pixels. The second affinity measure used in this study is color difference introduced in [16], where each pixel is treated as a vector in the RGB space and the difference between color angels is used to measure the similarity between two pixels:

$$w_{ij}^{c}=\text{exp}\left(\frac{-∠(I_{i}.I_{j})}{2\sigma_{c}^{2}}\right)$$ (8)

where σ_c_ represents variance between color difference of pixel *i* and its neighbor pixels. ∠ is the angle between pixel *i* and *j*. To obtain the affinity function, Eqs (7)) and (8) are combined as:

$$w_{ij}=w_{ij}^{I}w_{ij}^{c}$$ (9)

Based on the above explanation, the energy term for intrinsic image decomposition is formulated as:

$$E(R,S^{-1})=\underset{i\in I_{b}}{\sum }{\left(R_{i}-\underset{j\in \Omega_{i}}{\sum }w_{ij}R_{j}\right)}^{2}-\underset{i\in I_{b}}{\sum }{\left(I_{i}S_{i}^{-1}-R_{i}\right)}^{2}$$ (10)

where *Ω*_*i*_ is obtained by region growing *p*_*i*_. Fast region growing algorithm presented in [32] was employed for region growing. The optimization problem to be solved is then defined as:

$$\begin{array}{l} \;\underset{R,S^{-1}}{\text{arg min }E(R,S^{-1})}\;\forall_{i}\in I_{b}\\ subject\;to:\;0\leq R_{i,rgb}\leq 1 \end{array}$$ (11)

The quadratic function (11) was solved by setting the derivative of its dependent variables to zero (i.e.: *R*_*i*,*r*_, *R*_*i*,*g*_, *R*_*i*,*b*_, and *S*^*-1*^) and Gaussian-Seidel method was applied iteratively to improve the results of the linear solver [33].

## Discussion on selecting Ω

Selecting pixels to obtain the reflectance value at *p*_*i*_ needs to be handled carefully. In order to find the region *Ω*_*i*_, we performed region growing and used *p*_*i*_ as the input seed. Eq (9) was used to find *w*_*ij*_ for pixels in *Ω*_*i*_.

*Ω*_*i*_ obtained from region growing consists of pixels that more likely have the same reflectance. This is supported by Funt et al. [10] which suggests regions with similar reflectance to have similar chromaticity values. In contrast, Shen et. al. [7] selected pixels in a *k×k* patch centered at *p*_*i*_ to calculate the reflectance value at *p*_*i*_. This method does not impose any constraints on the properties of the selected pixel in the patch that limits the choice of *k* to small values. Thus, reflectance of each pixel is only represented by a limited number of neighboring pixels.

Our approach to finding the reflectance at *p*_*i*_ is also more advantageous compared to those methods that employ clustering or segmentation to find a region with similar reflectance to *p*_*i*_. As an example, Bi et al. [19] used clustering in the CIE-Lab color space and merged the generated clusters based on similarity measures to find regions with similar reflectance. It seems that their approach has two disadvantages: first, clustering generates unconnected image segments that require an additional merging step to find regions with similar reflectance. Second, selecting the efficient number of clusters for an arbitrary image is an issue on its own. An insufficient number of clusters leads to selecting clusters with high intra-class variance which in turn results in undesired cluster merging. Common issues such as handling empty clusters and algorithm convergence are other problems that need to be considered. The proposed region growing approach for finding pixels with similar reflectance ensures that *Ω*_*i*_ is connected and smooth.

Region growing is a costly process; therefore, we add two steps to speed up this process. First, to prevent generating very large regions, the region size is limited to pixels that are within a distance of *D* pixels from the input pixel (D = 30 was chosen in this study). Second, to execute the region growing algorithm less frequent, we make use of pixel labeling. For this task, let *Ω*_*last*_ be the last output of the region growing algorithm and *p*_*i*_ be the next input pixel. If *p*_*i*_ has been labeled to be part of *Ω*_*last*_, that is *p*_*i*_ ϵ *Ω*_*last*_, we let region *Ω*_*i*_ be the same as *Ω*_*last*_ with a small modification. To obtain *Ω*_*i*_, pixels farther than *D* pixels from *p*_*i*_ are removed from *Ω*_*last*_, and pixels closer than *D* with an intensity less than half of the average values of *Ω*_*last*_ are added to form *Ω*_*i*_. Algorithm 1 summarizes the steps preformed to generate the image region required to calculate *w*_*ij*_.

Algorithm 1. Reducing calls to region growing algorithm

1. Region distance = D;

2.  For input pixel *p*_*i*_

3.   If *p*_*i*_ ∉ *Ω*_*last*_:

4.    {Region Mask = 0

5.    Grow *Ω*_*i*_ until *||p*_*j*_*-p*_*i*_*||>D*

6.    Update *Ω*_*i*_};

7.   Else

8.    {from *Ω*_*last*_, remove pixels outside *||p*_*j*_*-p*_*i*_*||>D*

9.    For pixels in range *||p*_*j*_*-p*_*i*_*||*≤*D*

10.     If *p*_*j*_ *< average (Ω*_*i*_*)/2*

11.     Let *p*_*j*_ *ϵ Ω*_*i*_};

12.  Calculate *w*_*ij*_

### User brushes

In many applications such as texture mapping and matting, user interaction is required. This interaction can be extended to the intrinsic image decomposition process to improve the results. Bousseau et al. [6] recommended three kinds of user strokes for specifying local cues about illumination and reflectance. We apply the constant-reflectance and constant-illumination brushes. Pixels marked with constant-reflectance brush can be used to find a local cue about reflectance of the image pixels as:

$$B^{ref}(R)=\underset{i\in Ib}{\sum }z(\beta )\underset{j\in \beta_{i}^{ref}}{\sum }(R_{i}-R_{j})$$ (12)

where z(.) is a normalization factor defined as *z(β) = 1/| β|* that ensures strokes have an influence independent of their size, and | β| is the number of pixels specified by the user-brush. Eq (12) was generated taking R as the variable to be optimized since the optimization process was solved for reflectance and shading was obtained using Eq (5). The constant-illumination brush covering pixel *p*_*i*_ and *p*_*j*_ denotes that *I*_*j*_*S*_*j*_ = *I*_*i*_*S*_*j*_ and hence the energy function for this brush can be formulated as:

$$B^{lum}(R)=\underset{i\in Ib}{\sum }z(\beta )\underset{j\in \beta_{i}^{ref}}{\sum }(S_{i}J_{j}-S_{j}J_{i})$$ (13)

The optimization problem for intrinsic image decomposition with added user brushes can be defined as:

$$\begin{array}{l} \;\underset{R,S^{-1}}{\text{arg min }E(R,S^{-1})}+B^{ref}(R)+B^{lum}(R)\;\forall_{i}\in I_{b}\\ subject\;to:\;0\leq R_{i,rgb}\leq 1 \end{array}$$ (14)

**Fig 3:**
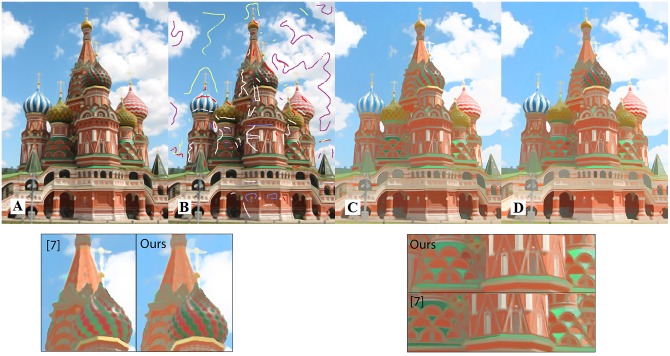
Reflectance component for the smooth input image with and without user brushes. (A) Original image, (B) image with added user brushes, (C, D) reflectance components without and with user brushes (S3 File).

Fig 3 shows the extracted reflectance component obtained with and without user brushes. In this figure, color lines identify regions with constant reflectance while the white lines show regions with constant shading. Magnified regions show how the effect of shadows has been decreased after implementing the user brushes.

### Learning material

After performing intrinsic decomposition for image *J*, the texture information should be assigned to the correct component. High frequency changes in the image are conventionally assigned to the reflectance component [8, 34]. However, there are cases that high frequency changes are part of the shading component (e.g. surfaces with bumps and wrinkles). In order to obtain better decomposition results, we apply material recognition which helps to assign texture information to the correct intrinsic component.

Material recognition is a challenging problem by itself and many methods have been proposed for solving this problem [18, 35, 36]. Bell et al. [18] presented a large scale dataset of materials in the wild and used learning techniques for material recognition. They enabled detection of materials in 23 categories which cover a large variety of materials (e.g. foliage, human skin, wood, glass and etc.). We directly use their implementation to find out if assigning texture information to intrinsic components based on material information has a positive effect on the intrinsic image decomposition problem.

**Fig 4:**
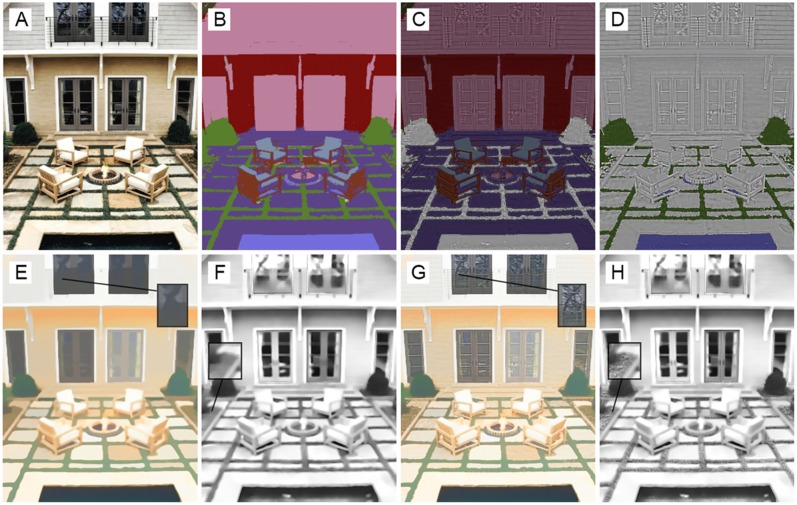
Assigning texture information to shading and reflectance components based on material of pixels. (A) Original Image, (B) result of material recognition. Colored regions in (C) and (D) show how texture should be assigned to reflectance and shading components respectively. (E) Reflectance obtained from *J*, (F) shading obtained from *J*, (G) final reflectance, and (H) final shading component (S4 File).

Fig 4 shows an example where material recognition was beneficial for adding texture details to the correct intrinsic component. Fig 4A and 4B show the reflectance and shading component obtained from the scene shown in Fig 2. The colored regions in Fig 4C and 4D show how the texture removed in the image smoothing process should be added to shading and reflectance components respectively. For example, in Fig 4D, the foliage and water are the colored regions and hence, the texture details of pixels that belong to these regions should be added to the shading component. Fig 4E–4H show the intrinsic components before and after assigning texture components to shading and reflectance images. It can be observed that fine details added to the shading and reflectance images improve the results. A Lookup Table (LUT) was created for assigning texture details to the intrinsic components. A number between 1 and 23 was assigned to each unique material in the first column of the LUT. The second column of the LUT determines the intrinsic component for the corresponding material. For example, the texture details of Foliage were assigned to the shading component and the texture details of materials such as Brick, Fabric, and Carpet were assigned to reflectance component. After detecting the material type for an input pixel, the texture details of that pixel were assigned to the reflectance or shading component based on this LUT. The correspondence between each material class with shading and reflectance components is supplied in the supplementary material (S1 File).

## Experimental Results

**Fig 5:**
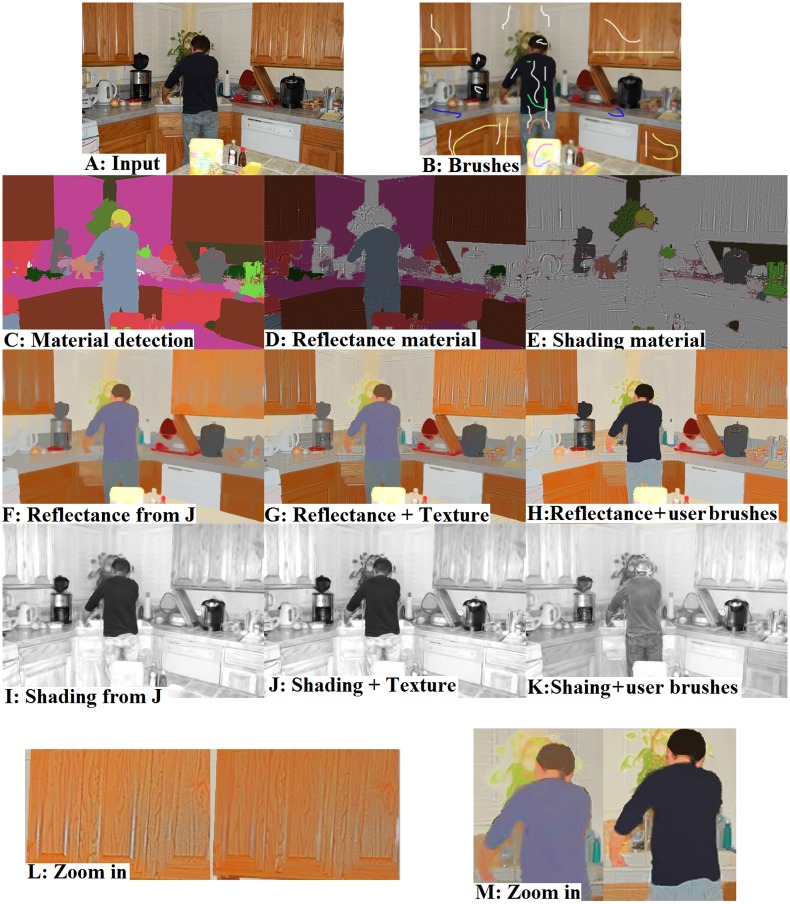
Illustration of the intrinsic image decomposition pipe-line. (A) Original image, (B) smooth image with added user brushes, (C) material segmentation, (D) reflectance material, (E) shading material, (F) reflectance component obtained from *J*, (G) reflectance component with added texture detail, (H) reflectance component obtained by applying user brushes, (I) shading component of the smooth image, (J) shading component with added texture details, and (K) shading component with added user brushes (S5 File).

This section reports the experimental results to evaluate the performance of the proposed method. The full pipe-line for producing the intrinsic components is illustrated in Fig 5. The input image and its smooth version (*J*) are shown in the top row. The added user brushes are also depicted in the smooth image. Fig 5C shows the material segmentation step, where each color indicates a unique material. Fig 5D and 5E illustrate how texture information should be added to the reflectance or shading components. Fig 5F and 5I illustrate the extracted reflectance and shading components for the smooth image, while Fig 5G and 5J show the reflectance and shading components with added texture details. As an example, texture of the human hair, shown in yellow, was added to the shading component (Fig 5J), while the texture information of the purple region was assigned to the reflectance component (Fig 5I). To our knowledge, this is a very fine-level improvement in intrinsic image decomposition since previous methods do not provide such solutions for handling small illumination variations. Fig 5H and 5K show the reflectance and shading components when user brushes were considered in the decomposition process. The texture details were added the same as the automatic decomposition mode.

**Fig 6:**
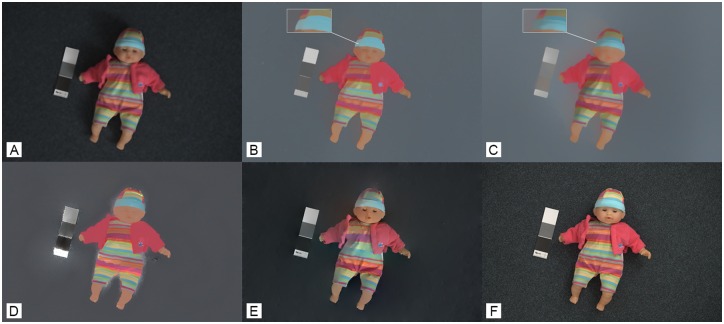
An illustrative comparison between image decomposition results using are method and methods of [6, 7, 16, 37]. (A) The original image. Decomposed reflectance component by: (B) our method, (c) Shen et al. [7], (d) Bousseau et al. [6], (E) Tappen et al. [16], and (F) Weiss at al. [37] (S6 File).

Fig 6 shows the performance of the proposed method for handling non-smooth shading without applying user brushes (i.e. the doll’s winter cap). The figures illustrate improvement of results when compared with the methods of [6, 7, 16, 37]. This improvement is due to texture removal in the first stage of the proposed algorithm which enables more accurate selection of regions with similar reflectance. As previously discussed, Shen et al. [8] used a small patch of pixels for reflectance calculation. This has led to obtaining different reflectance values for a region with the same material, as shown in Fig 6. In [6], the effect of JPEG compression significantly distorts the output reflectance. In [16] and [37] the reflectance components are not correctly extracted and lots of shading details are mistakenly added to the reflectance image.

**Fig 7:**
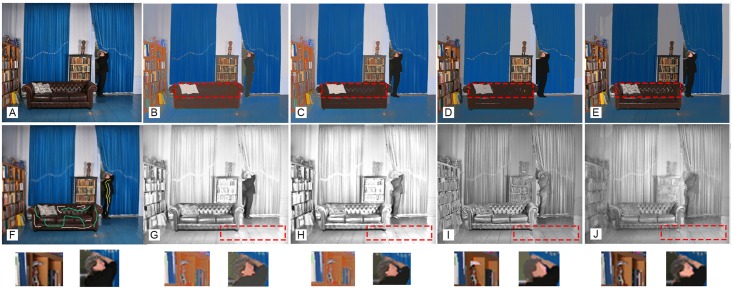
Intrinsic image decomposition results from our method and methods of [19] and [38]. (A, F) The original image and added user brushes, (B, C) reflectance components without and with user brushes, (D) reflectance component obtained in Bi et al. [19], (E) reflectance component obtained in Bell et al. [38]. (G, H) shading component from our method without and with user brushes, (I) shading component obtained from Bi et al. [19] and (J) shading component obtained from the method of Bell et al. [38] (S7 File).

Fig 7 shows a visual comparison between our method and recent methods of [19] and [38] which adopt the sparsity assumption on reflectance. The input image is quite complex due to large shading variations such as highlights and shadows (Fig 7A). Fig 7F shows the input image with added brushes. Fig 7B–7D show the reflectance components extracted from our method and methods of [19] and [38], respectively. Three regions are shown distinctly for better visualization. First, the reflectance component for the human face region extracted using our method is more uniform compared to the results of [19, 38]. Second, our method was able to handle shading component in the sofa region more accurately and specular regions were correctly added to the shading component. Third, the shading component of the floor region is correctly extracted by our method, where the effect of shadow and light reflectance were clearly added to the shading component.

**Fig 8:**
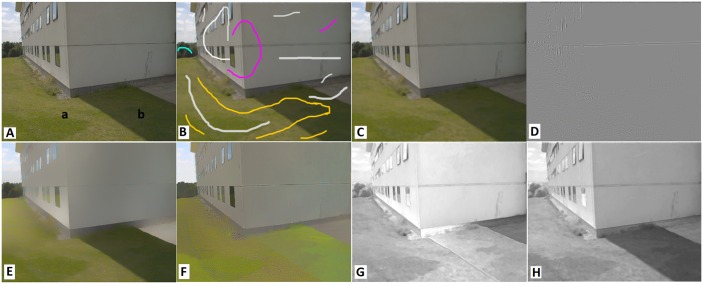
Handling intense lighting conditions with user brushes. (A) Original image, (B) user brushes, (C, D) intrinsic components with and without user brushes (S8 File).

In Fig 8 we illustrate how our method can handle intense lighting conditions. The building in the scene has produced an intense shadow region on the grass leading to a large color difference between regions “a” and “b”, which cannot be handled with region growing introduced in (6). Fig 8B shows the user brushes applied to the image which specify regions with constant shading or reflectance. Fig 8E and 8F show the extracted shading and reflectance components with and without user brushes. When brushes were applied the shadows on the grass were correctly detected and assigned to shading component.

**Fig 9:**
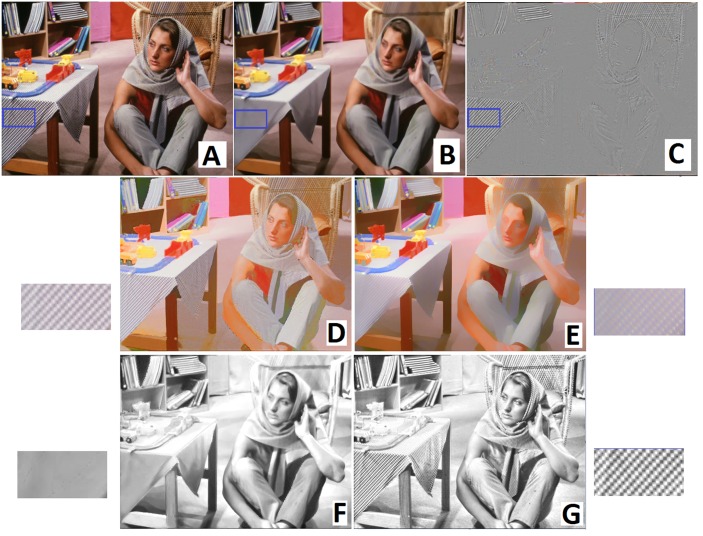
Effect of image smoothing in the intrinsic image decomposition. (A) Original image, (B) smooth image, (C) texture image, Images (D,E,F,G) show the reflectance and shading components when using our method and Shen et al. [7] respectively (S9 File).

Fig 9 shows an example were processing the smooth image for intrinsic image decomposition is beneficial. This example is focused on the patterned fabric. We compare the result of our method with that of Shen et al. [7], where the raw input image is processed for intrinsic decomposition. It is obvious that the pattern of the fabric is part of the reflectance component. Fig 9A shows the original image and Fig 9B and 9C show the structural and texture components. The result of intrinsic decomposition using our method and Shen et al. [7] are shown in Fig 9D through 9G. Using our method, the texture details of the fabric region were correctly assigned to the reflectance component. This example shows that our method can be used for intrinsic image decomposition of images with fine texture details.

For quantitative evaluation of our intrinsic image decomposition method, ground truth data about the scene geometry and lighting conditions is required. Intrinsic images in the wild and the MIT intrinsic image dataset are the available datasets that represent images in terms of their intrinsic components. The intrinsic images database is a collection of annotated images from real-world consumer photographs. In this database, surface segments are drown on crowd souring and surface properties including textural and contextual information are added for each segment [18]. The MIT intrinsic images dataset was created by Grosse et al. [15]. To create the complete image, each object was photographed using a polarizing filter set to maximum specular value. The diffuse image was captured by setting up the filter to remove specular regions. Each object was then painted and re-captured to obtain the shading component. These three images were used to calculate the reflectance and specular images. We used the MIT dataset since special imaging conditions were considered for acquiring the shading and reflectance components. Further explanation about the MIT intrinsic dataset can be found in [15]. The objective performance measures used in this evaluation was the Least Mean Square Error (LMSE) as defined in [15].

Table 1 provides the LMSE values obtained from our method with and without applying user brushes. The results presented by Shen J. et al. [7], Shen L. et al. [22], and Color-Retinex [39] as reported in [15] are also shown in Table 1. The values shown in bold indicate the lowest value for each image among the tested methods. For method of Shen J. et al. [21] and Color-Retinex, the LMSE values for images that contain specular regions were not provided. The values in Table 1 show that when user brushes were applied, the LMSE values for our method were less than the previous methods except for Teabag1 and Teabag2. We have calculated the average value of LMSE values for all the images except those with specular regions. The average value for our method with user brushes was 0.0129 which is about 0.07 better that the method of Shen L, et al. [22]. LMSE values for images with specular regions were obtained by adding the ground truth shading and specular values.

**Table 1:** Comparison of LMSE values of our results and the results by previous works [7, 22, and 39] on the MIT dataset.

	Our method without brushes	Our method with brushes	Shen J. et al.[7]	Shen L. et al.[22]	Finlayson GD et al. [39]
Box	0.0061	**0.0051**	0.0065	0.0018	0.013
Cup 1	0.0054	**0.0023**	0.0057	0.0030	0.007
Cup 2	0.0068	**0.0035**	0.0892	0.0045	0.011
Deer	0.0418	**0.0341**	0.0312	0.0419	0.041
Dinosaur	0.0212	**0.0183**	0.0228	0.0216	0.035
Frog 1	0.0523	**0.0424**	0.0783	0.0482	0.066
Frog 2	0.0067	**0.0241**	0.0094	0.0472	0.071
Panther	0.0943	**0.0068**	0.0087	0.0078	0.011
Paper 1	0.0334	**0.0011**	0.0385	0.0014	0.004
Paper 2	0.0031	**0.0019**	0.0034	0.0021	0.004
Raccoon	0.0041	**0.0035**	0.0044	0.0048	0.015
Squirrel	0.0037	**0.0035**	0.0041	0.079	0.072
Sun	0.0025	**0.0018**	0.0027	0.0023	0.003
Tea bag 1	0.039	0.034	0.0410	**0.0280**	0.032
Tea bag 2	0.0291	0.024	0.0320	**0.0141**	0.023
Turtle	0.0019	**0.0018**	0.0210	0.0174	0.069
Apple	0.008	**0.0056**	0.0077	-	-
Pear	0.0039	**0.0034**	0.0045	-	-
Phone	0.0043	**0.0039**	0.0059	-	-
Potato	0.0045	**0.0032**	0.0054	-	-
Average	0.0219	**0.0129**	0.0249	0.0203	0.0298

**Fig 10:**
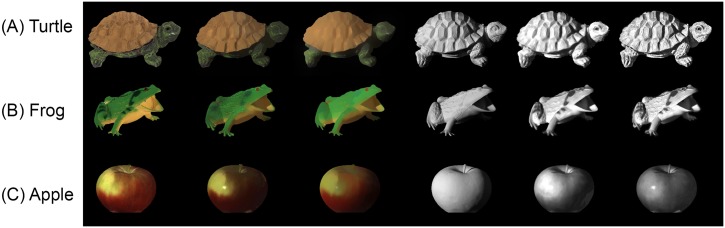
Example of reflectance and shading components extracted from the MIT intrinsic image dataset. For each example the following are shown in order: Original image, ground truth reflectance, reflectance component from our method, reflectance component from Shen et al. [7], ground truth shading component, shading component obtained from our method, shading component obtained from Shen el al. [7].

Fig 10 illustrates the shading and reflectance components for three examples from the MIT dataset without using user brushes. For each image, the ground truth reflectance and shading components are also shown. For visual comparison, the output reflectance and shading components obtained from the work Shen J. et al. [7] are also depicted. The first row shows the shading and reflectance components for the turtle image. This example illustrates the effectiveness of the proposed method approach to handle the texture. Our method has successfully extracted the reflectance component while in the reflectance component extracted from [7], the reflectance details are incorrectly added to the shading component. In the second row, the example shows that our reflectance output is more monotonic compared to the reflectance component obtained from the work of Shen J. et al. [7]. The last row shows a limitation of the proposed method when user brushes were not applied. For this image, the surface of the apple violates the Lambert assumption, and the reflectance component of this image contains smooth reflectance variations. The complete results on the MIT intrinsic images dataset including Base, Shading, Reflectance, and Texture images can be found in (S10 File).

**Fig 11:**
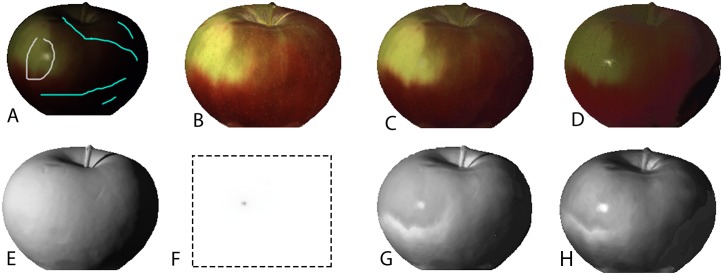
Results of intrinsic image decomposition for the image of the Apple, with and without user brushes. (A) Original image with added user brushes, (B) ground truth reflectance, (C) reflectance with user brushes, (D) reflectance without user brushes, (E, H) ground truth shading and specular components, (G) shading with user- brushes, and (H) shading without user brushes.

In order to illustrate how the added user brushes improve the output of our algorithm, the result of intrinsic decomposition for the image Apple from the MIT dataset is shown in Fig 11. Reflectance and shading components extracted with and without user brushes, added user brushes, and the ground truth images are shown in this figure. When user brushes were applied, more shadows have been correctly assigned to the shading component. Also, the specular region on the surface of the apple were correctly assigned to the shading component.

**Fig 12:**
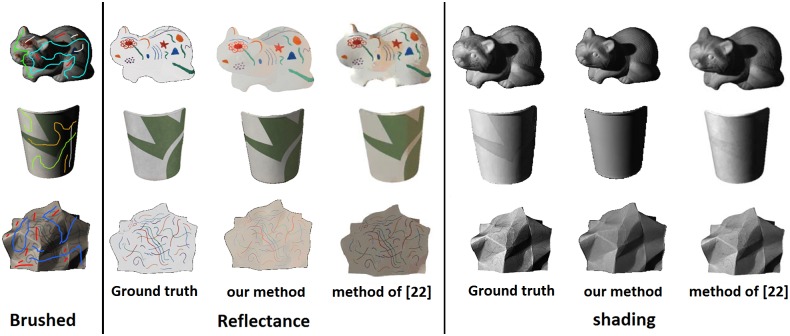
Result of intrinsic image decomposition by applying user brushes. Images in the left column show how the brushes were applied. Images in the second section show the reflectance components and the third section shows the shading components.

Fig 12 shows more examples of intrinsic image decomposition using our method and the method of Shen L. et al. [22]. For the image Raccoon, applying the constant reflectance brush resulted in accurate extraction of the reflectance component. In addition, the shadows in the original image were correctly added to the shading component. For Cup1, less shading information is present in the reflectance component compared to Shen L. et al. [22], however, the shading component from Shen L. et al. [22] method holds less reflectance information. The image of the Paper1 is another example with complex shading. As the results shows, our method has been able to correctly decompose this surface into shading and reflectance components.

### Implementation

Our algorithm was implemented in MATLAB 2015a using an Intel Corei7 CPU for data processing. The region growing algorithm was programmed in C++ and combined with the rest of the code through a Mex file. For image smoothing, the code provided by Karacan et al. [27] was utilized. Our intrinsic decomposition was implemented in two parts: weight matrix calculation and optimization step. Calculating the *w*_*ij*_ matrix required less than 1 minute to process for an image with 640×480 pixels. The Gaussian- Seidel iterative process requires intensive vector multiplications and each iteration takes about 10–13 second. In general, 15 to 30 iterations were required to obtain the reported results. The time variation in the optimization step for each iteration was due to un-fixed *w*_*ij*_ vector length for each pixel. The vector size was determined by the number of pixels in *Ω*_*i*_. The overall intrinsic decomposition processing time of our approach is comparable to recent intrinsic image decomposition algorithms. Bi et al. [19] has reported that their image decomposition pipeline requires about 5 to 10 minutes to process. The proposed method by Shen J. et al. [7] requires 200 iterations for the algorithm to obtain acceptable decomposition, and each iteration requires 2 to 5 seconds of processing time depending on the size of image patches used for calculating the weights. Weight calculations should also be added to the processing time required for intrinsic decomposition for this method. Parallel processing and utilization of GPU can improve the computation speed of our algorithm, which is also a recommended solution suggested in [7, 19].

## Conclusions

A framework for intrinsic image decomposition was presented in this paper. Our method was based on the assumption that pixels in a region with similar chromaticity share the same reflectance value. To ensure Mondrian-like images, we applied structure preserving image smoothing and processed the smooth image for intrinsic decomposition. The reflectance component was obtained by minimizing an energy function which defined the reflectance value of a pixel as the weighted sum of reflectance values of pixels obtained by region growing the current pixel. Texture details separated in the smoothing step were added to the shading or reflectance components based on the material of each pixel. Qualitative examples showed that processing the smooth image along with user brushes and material recognition results in correct separation of shading and reflectance components for a wide range of images with large illumination variations and complex surfaces. Quantitative experiments conducted on the MIT dataset showed that our method has improved the quality of intrinsic image decomposition compared to previous methods tested on this dataset. The average LMSE values of our method on the MIT intrinsic images was about 0.013 which showed to be at least 0.07 better than the methods tested on this dataset. Altogether, our approach to intrinsic image decomposition allows for accurate extraction of shading and reflectance components for a wide range of images which makes this method attractive for applications that require intrinsic components of the image.

## Supporting Information

S1 File Correspondence between Materials and Intrinsic components. (RAR)

S2 File Full-size images in Fig 2. (RAR)

S3 File Full-size images in Fig 3. (RAR)

S4 File Full-size images in Fig 4. (RAR)

S5 File Full-size images in Fig 5. (RAR)

S6 File Full-size images in Fig 6. (RAR)

S7 File Full-size images in Fig 7. (RAR)

S8 File Full-size images in Fig 8. (RAR)

S9 File Full-size images in Fig 9. (RAR)

S10 File Results of our method on MIT intrinsic images data set. (RAR)
