# Correction: Intrinsic Image Decomposition via Structure-Preserving Image Smoothing and Material Recognition

**DOI:** 10.1371/journal.pone.0171949

**Published:** 2017-02-06

**Authors:** Ali Nadian-Ghomsheh, Yasin Hasanian, Keyvan Navi

The second author's name is spelled incorrectly. The correct name is: Yasin Hasanian. The correct citation is: Nadian-Ghomsheh A, Hasanian Y, Navi K (2016) Intrinsic Image Decomposition via Structure-Preserving Image Smoothing and Material Recognition. PLoS ONE 11(12): e0166772. doi:10.1371/journal.pone.0166772
